# Healing the unhealable: Wharton's jelly stem cell vesicles as a breakthrough for feline chronic skin ulcers

**DOI:** 10.1186/s13620-026-00341-7

**Published:** 2026-04-14

**Authors:** Mohamed S. kishta, Salem A. Serag, Ahmed N. Abdallah, Mohamed M. Bahr, Ashraf A. Shamaa, Abdallah M. Hafez, Basma Salah

**Affiliations:** 1 Hormones Department, Medical Research and Clinical Studies Institute, National Research Centre, P.O.B: 12622, 33 El Buhouth St., El Dokki, Cairo, Egypt; 2 Stem Cell laB, Center of Excellence, National Research Centre, Cairo, Egypt; 3 London Pet CliniC, Cairo, Egypt; 4 Surgery, Anesthesiology and Radiology Department, Faculty of Veterinary Medicine, Cairo University, Cairo, Egypt; 5 Medical Biochemistry Department, Faculty of Medicine, Kafrelsheikh University, Kafr Elsheikh, Egypt; 6 Department of Anatomy and Embryology, Faculty of Medicine, Kafrelsheikh University, Kafr El-Sheikh, Egypt

**Keywords:** Extracellular vesicles, Clinically non-healing ulcer, Wound, Wharton Jelly, Mesenchymal stem cells, Cats

## Abstract

Chronic skin wounds in companion animals, particularly cats, often persist due to a combination of impaired healing responses and underlying primary etiologies, such as trauma, post-surgical dehiscence, pressure ulcers, allekirgic dermatitis, or immune-mediated conditions. Effective management therefore requires both accurate diagnosis and treatment of these primary causes and the implementation of strategies to promote tissue repair and regeneration. While an acute wound typically heals within 4–6 weeks through orderly phases of hemostasis, inflammation, proliferation, and remodeling, a chronic wound remains unresolved beyond this period, often due to prolonged inflammation or disruption of one or more healing stages. This study evaluated the therapeutic efficacy of WJ-MSC-derived EVs in adult cats with chronic ulcers persisting for more than four weeks. These EVs support essential biological processes, including immune modulation, tissue regeneration, cellular homeostasis, anti-inflammatory actions, and anti-fibrotic activity. Twenty cats were divided into two groups: an EV-treated group receiving carboxymethyl cellulose (CMC) gel loaded loaded with Wharton's Jelly mesenchymal stem cell-derived extracellular vesicles (WJ-MSC-derived EVs) (100 μg EV protein/cm^2^ wound area) alongside standard care, and a control group receiving CMC gel (in house prepared 2% concentration) alone. EV treatment significantly accelerated ulcer healing, with markedly enhanced wound contraction and near-complete closure by Day 16 (92.4 ± 6.8%) compared with controls (58.3 ± 12.1% at Day 16). Histopathology revealed superior epithelialization, earlier collagen deposition, and better connective tissue organization in EV-treated wounds. Immunohistochemistry demonstrated increased α-SMA and CD31 expression, indicating active angiogenesis and tissue remodeling. Additionally, scratch assays showed improved cellular migration in response to EVs. Overall, In this pilot study of client-owned cats, WJ-MSC-derived EVs demonstrated beneficial effects on wound healing, with significantly accelerated contraction and improved histological organization compared to standard care alone. However, due to the small sample size, etiological and therapeutic heterogeneity, and study design limitations, external validity remains limited. These findings suggest WJ-MSC-derived EVs may represent a promising adjunctive therapy for feline chronic ulcers, but larger, etiology-stratified trials are needed before recommending this as an established alternative treatment. Further research should focus on standardizing treatment protocols, identifying optimal patient selection criteria, and evaluating long-term outcomes.

**Supplementary Information:**

The online version contains supplementary material available at 10.1186/s13620-026-00341-7.

## Introduction

Chronic skin ulcers represent a significant clinical challenge in veterinary medicine, particularly in companion animals such as cats and dogs [1]. Effective management therefore requires both identification and control of underlying causes and advanced strategies to promote healing [2]. Unlike in human medicine, where chronic wounds are frequently associated with systemic conditions like diabetes mellitus, peripheral vascular disease, or prolonged pressure, chronic wounds in veterinary patients more commonly arise from trauma, surgical complications, infections, foreign bodies, or underlying allergies and immune-mediated disorders. Although the overall economic burden in veterinary practice may be lower proportionally than in human healthcare due to differences in treatment costs, shorter patient lifespans, and varying owner investment, these non-healing wounds nonetheless impose substantial financial and emotional costs on pet owners, often leading to prolonged treatment, repeated veterinary visits, and, in severe cases, euthanasia. In cats, chronic skin ulcers frequently result from persistent self-trauma secondary to pruritus (e.g., flea allergy dermatitis, food hypersensitivity, atopic dermatitis), neuropathic pain, unresolved or recurrent infections (often bacterial or mixed, secondary to the above), traumatic injuries with complications, or post-surgical dehiscence. These factors sustain prolonged inflammation and impair progressive healing despite standard care [3].

Mesenchymal stem cells have a natural ability to travel to injured areas[4, 5], calm inflammation, and promote the healing process by releasing a mix of signals like chemokines, cytokines, and growth factors that help cells grow and repair damaged tissue [6, 7]. Primarily by paracrine activity, stem cells promote wound healing by producing extracellular vesicles (EVs), which are potent repair mediators [8, 9]. These MSC-derived EVs carry a complex cargo of nucleic acids, proteins, and lipids, enabling efficient communication between cells [10], EVs are essential for accelerating the healing of wounds and encouraging the creation of scars because they facilitate these interactions [11, 12]. It has been demonstrated that EVs derived from human umbilical cord MSCs specifically promote blood coagulation [13], a crucial function in the early hemostatic phase of wound repair. By inducing vasoconstriction and promoting platelet aggregation, they successfully minimize blood loss during this phase, setting the way for later healing phases [8].

Mesenchymal stem cell-derived extracellular vesicles (MSC-EVs) modulate macrophage polarization [14]. They promote the transition from pro-inflammatory M1 macrophages to anti-inflammatory and pro-reparative M2 macrophages, which is marked by the release of Interleukin-1 Beta and Tumor Necrosis Factor Alpha. When the macrophages are in this repair phase, they emit calming signals like TGF-β and IL-10, which reduce inflammation and indicate tissue healing. As a result, the MSC-EVs are facilitating the immune system to cease fighting and begin healing [15]. It's interesting to note that MSC-EVs display a unique mix of surface markers, including mesenchymal stem cell signature antigens like CD44, CD73, and CD90 together with usual extracellular vesicle antigens like CD63, CD9, and CD81 [16, 17].

It has been discovered that MSC-EVs activate the PI3K/AKT pathway, which is essential for wound healing [18–20]. Because of this activation, TGF-β1 and fibronectin are expressed more, which together improve epithelialization and promote fibroblast migration and proliferation. This increases the secretion of type I and type III collagen, Matrix Metalloproteinase-1 (MMP-1), and other ECM-related proteins that are essential for tissue regeneration and repair [5, 12, 21]. Platelet-Derived Growth Factor (PDGF), Epidermal Growth Factor (EGF), Fibroblast Growth Factor (FGF), TGF, Placental Growth Factor (PlGF), Insulin-Like Growth Factor (IGF), and most significantly, vascular endothelial growth factor (VEGF), are among the several proangiogenic factors that MSCs make and which can control angiogenesis [22–25]. Additionally, they alter the angiogenesis indicators' cell surface proteins, such as Platelet Endothelial Cell Adhesion Molecule (PECAM-1)/CD31 [26, 27]. Additionally, the induction of MSCs into the transformation of contractile myofibroblasts (MFs) by tissue repair and the tumor microenvironment develops new stress fibers containing α-smooth muscle actin (α-SMA) [28]. Furthermore, myofibroblasts express extracellular matrix and are responsible for the contraction in granulation tissue, where α-SMA is presented as a specific indicator representing myofibroblast cells [29, 30]. Wharton's Jelly (WJ) is classified as a connective tissue that provides structural stability to the umbilical cord. Its main components include collagens, glycosaminoglycans, and proteoglycans, which together form an extracellular matrix rich in healing attributes [31]. WJ is abundant in MSCs and can be obtained with much ease compared to other sources, as it is normally cut without harming the donor and then discarded [32, 33]. The healing effect on chronic ulcers lasting beyond four weeks using EVs collected from WJ-MSCs is unexplored. Despite promising preclinical evidence supporting the regenerative potential of MSC-derived extracellular vesicles (EVs) in various mammalian wound models, their therapeutic efficacy in naturally occurring chronic skin ulcers in companion animals remains largely unexplored. This knowledge gap prompted the present study with the following research question: Can local administration of Wharton’s Jelly mesenchymal stem cell-derived EVs accelerate healing and improve tissue regeneration in feline chronic skin ulcers that are refractory to standard care?

## Materials and methods

### Reagents

Collagenase VI, phosphate-buffered saline (PBS), culture medium, RPMI medium, bovine serum albumin were all purchased from (BSA, SigmA, USA), medium 199 with 25 mM HEPES, Beckman Coulter Optima L 90 K ultracentrifuge, anti-CD31 antibody were all purchased from (Abcam, United Kingdom), HRP-coupled secondary antibody were purchased from (Aspen, China), and diaminobenzidine (DAB).

### WJ-MSCs isolation and characterization

**Fig. 1:**
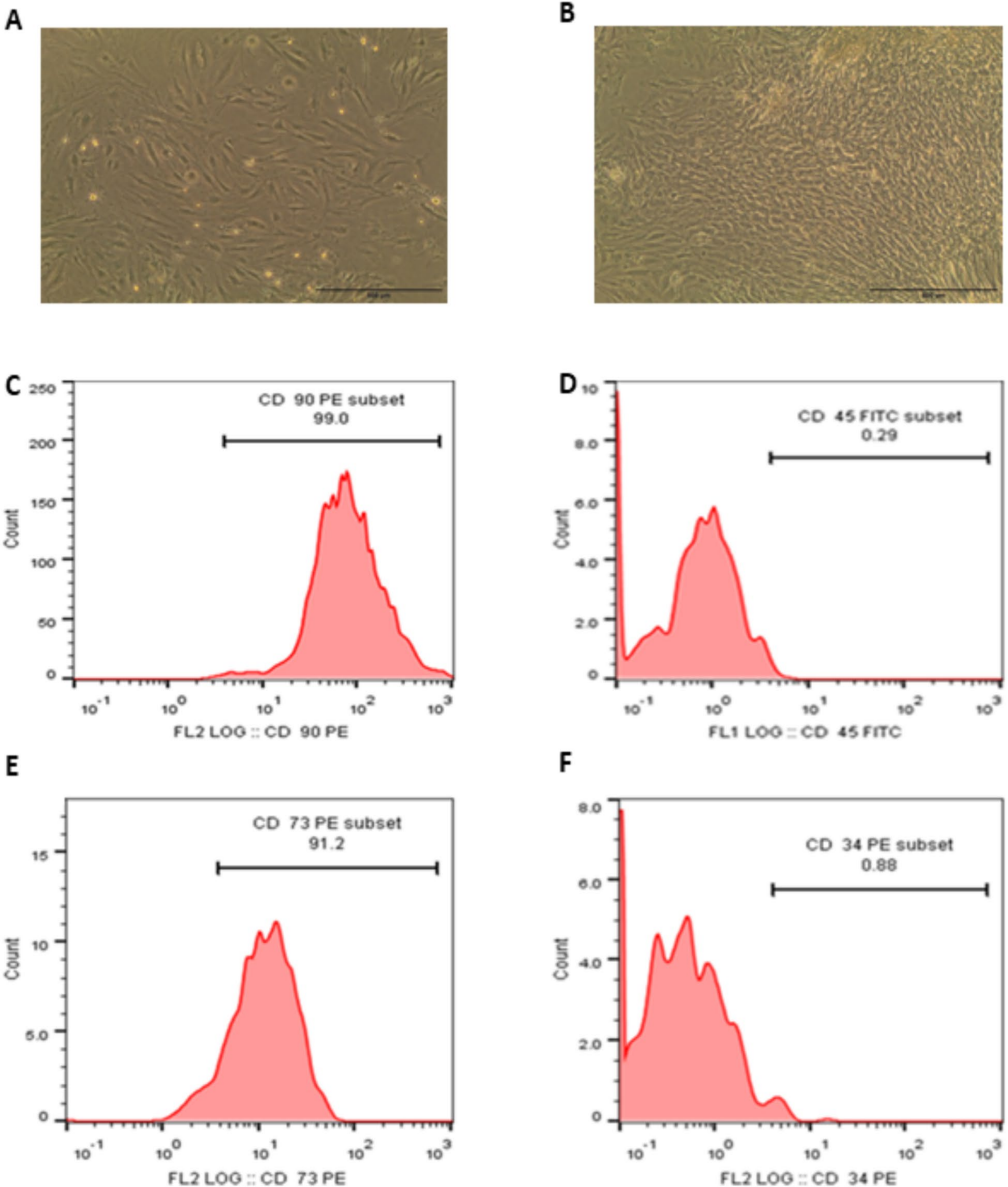
Characterization of Wharton Jelly Mesenchymal Stem Cells (WJ-MSCs) by morphological and flow cytometric analysis. **a** Cells at 70% confluence showing typical spindle shape; **b** cells at complete confluence forming a monolayer. Flow cytometry analysis revealed high expression of CD90 (99%) in (**c**), minimal expression of CD45 (0.29%) in (**D**), high expression of CD73 (91.2%) in (**e**), and low expression of CD34 (0.88%) in (**f**), indicating MSC identity

A sample of Wharton’s Jelly tissue weighing two grams was minced into 1–2 mm pieces and incubated in culture medium for seven days in a 100-mm culture dish. Collagenase VI (2 mg/ml) was then used to enzymatically degrade the tissue pieces in a 15 ml tube at 37 °C for one and a half hours. Following digestion, the solution was filtered to exclude undigested tissue, centrifuged at 200 × g, and then rinsed three times with phosphate-buffered saline (PBS). The cells were then resuspended in 10 ml of culture media after being centrifuged at 700 × g. They were then incubated in a flask at 37 °C with 5% CO₂, with the medium being changed every three days. An inverted microscope was used to record spindle-shaped and flattened cells once cell confluence was achieved (Fig. 1a and b). The cells' morphology and flow cytometry analysis were assessed using the markers CD34, CD45, CD73, and CD90.

### Isolation of the WJ-MSCs EVs

The supernatants of stem cells at the fourth passage (5 × 10⁶ cells/ml) were cultivated in Roswell Park Memorial Institute (RPMI) medium devoid of fetal bovine serum (FBS) and supplemented with 0.5% bovine serum albumin (BSA, SigmA, USA) to extract extracellular vesicles (EVs). After centrifuging the supernatants for 20 min at 2000 × g to eliminate debris, they were ultracentrifuged for an hour at 4 °C at 100,000 × g using a Beckman Coulter Optima L 90 K ultracentrifuge. After being cleaned in serum-free medium 199 with 25 mM HEPES (SigmA, USA), the supernatants were subjected to a second ultracentrifugation under the same circumstances. The Bradford technique (BioRad, Hercules, CA) was used to determine the protein content of the EVs, and samples were kept in collecting medium at −80 °C until they were needed again.

### Characterization of WJ-MSCs EVs

**Fig. 2:**
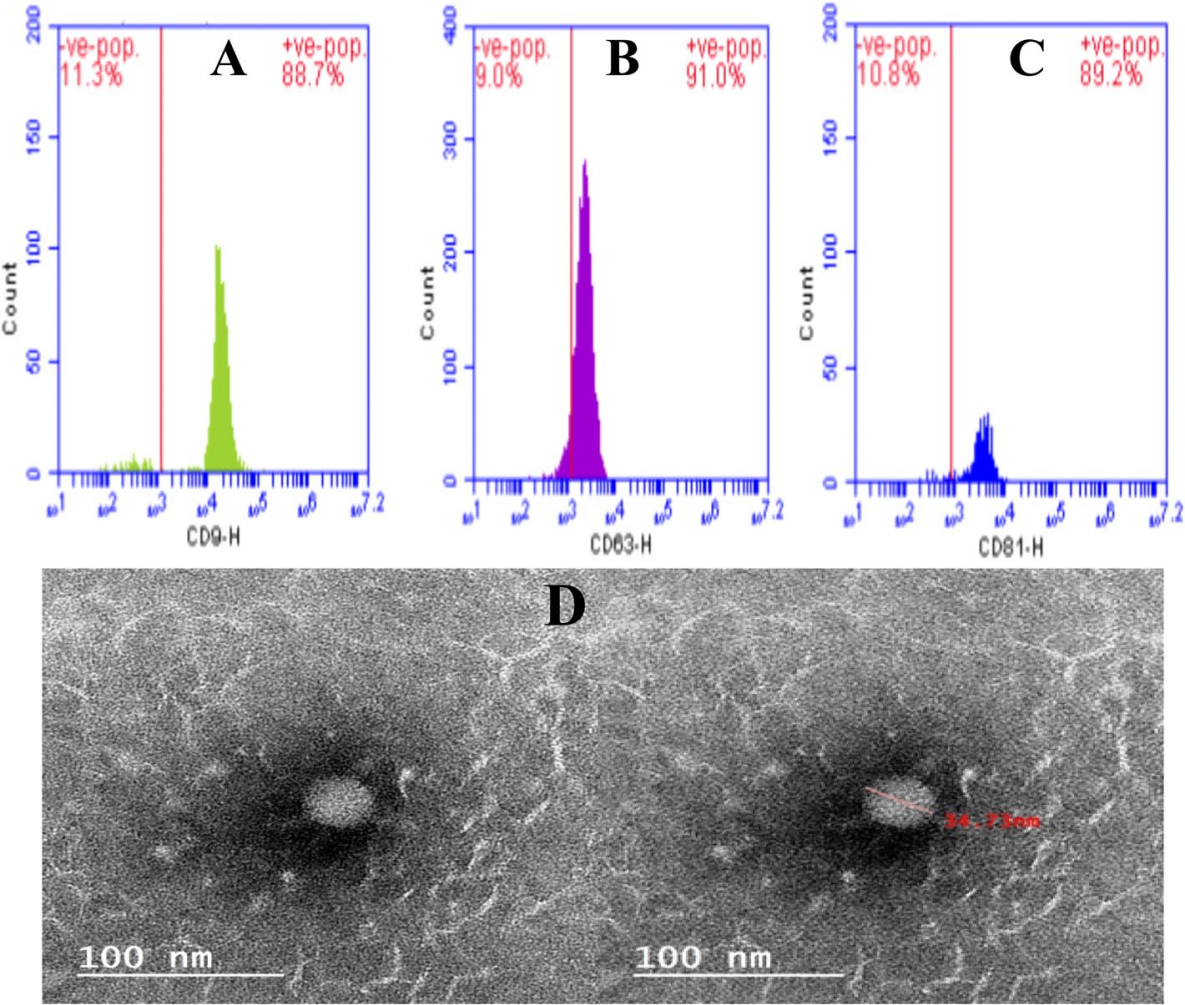
Characterization of Wharton’s Jelly MSC-derived extracellular vesicles (WJ-MSC EVs) and wound healing assay. **A**, **B**, **C** Flow cytometry analysis demonstrated high expression of WJ-MSC EV surface markers CD9 (88.7%), CD63 (91.0%), and CD81 (89.2%), indicating the purity and identity of the isolated EVs. **D** Transmission electron microscopy (TEM) reveals the typical spherical morphology and nanoscale size (34.73 nm) of EVs with intact membrane structures, confirming the successful isolation of functional vesicles

As seen in Figs. 2B, C, D, and F, EVs were described using flow cytometry to further illustrate the protein expression on the surface of CD9, CD63, and CD81 of the EVs produced from Wharton's Jelly mesenchymal stem cells (WJ-MSC). Isolated WJ-MSC extracellular vesicles were fixed and mounted onto Formvar-coated copper grids. After adsorption, grids were washed, negatively stained, and air-dried. Samples were examined using a transmission electron microscope to assess vesicle morphology and size. Image analysis was performed using appropriate imaging software.

### Wound healing (or scratch) assay

**Fig. 3:**
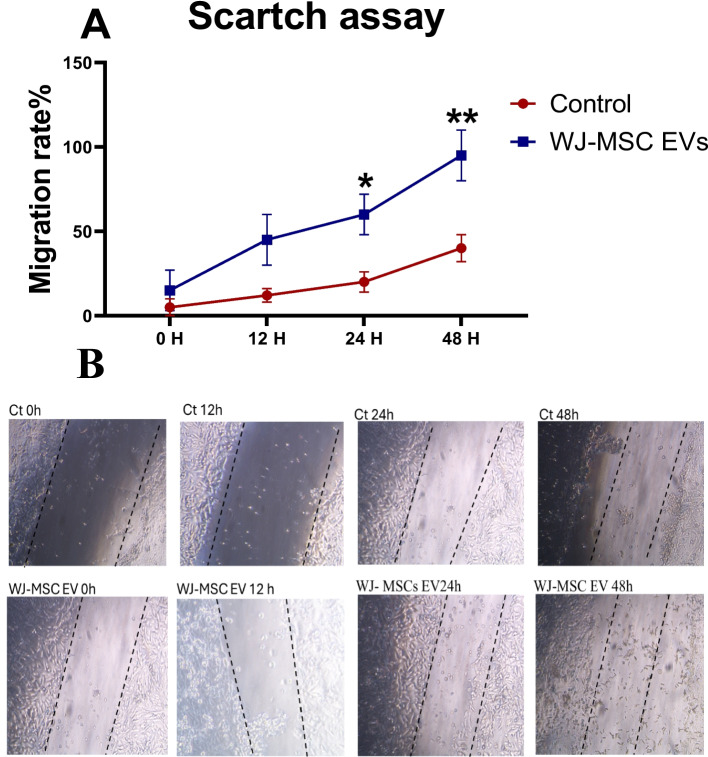
(**A**) Scratch wound healing assay showing enhanced cell migration in the WJ-MSC EV-treated group compared to Control across 0, 12, 24, and 48 h, confirming their pro-regenerative effect. Statistically significant differences between the EV-treated and Control groups.*P < 0.01 at 24 h and **P < 0.001 at 48 h. **(B)** Compares cell migration rates between the control group and the WJ-MSC EV-treated group over 0, 12, 24, and 48 h. In the control group, cell migration increased gradually from 5% at 0 h to 15%, 20%, and 35% at 12, 24, and 48 h, respectively. In contrast, the WJ-MSC EV-treated group demonstrated significantly enhanced migration, starting at 10% at 0 h and increasing to 30%, 40%, and 60% at 12, 24, and 48 h, respectively

To assess EVs' capacity to promote cell migration, a scratch wound experiment was also carried out. Mesenchymal stem cells were plated on a 24-well cell culture plate for this experiment, and a 200 μL pipette tip was used to make a straight line in the cell monolayer, creating a scratch. The cells were then incubated at 37 °C after 500 μL of either phosphate-buffered saline (PBS) or Wharton's Jelly mesenchymal stem cell (WJ-MSC) extracellular vesicles (EVs) were introduced to the corresponding wells. Using an inverted microscope, the gap distance was tracked and measured statistically. As shown in Figs. 2c and Fig. 3, the migration rate was computed using the following formula: Migration Rate (%) = (L0—Lt)/L0 × 100%, where L0 is the starting scratch width and Lt is the scratch width at a given time t.

### Animals

#### The study design

This study was designed as a prospective, randomized, double-blind, controlled clinical trial comparing WJ-MSC-derived EV therapy with standard care in client-owned cats with chronic ulcers.

We employed 20 adult cats of both sexes, weighing between 5 and 10 kg, who had chronic ulcers (ulcers that lasted longer than four weeks). At the London Pet Clinic, the animals were kept in appropriate individual cages with free access to water and typical cat food. A 12-h dark–light cycle, a regulated temperature of 20 ± 2 °C, and suitable thresholds for ambient relative humidity and noise were all maintained. The Ethics Committee on Animal Use at Kafr Elsheikh University approved this research (Approved protocol KFS-IACUC/207/2024) All cats were client-owned animals, and written informed consent was obtained from all owners prior to enrollment. Owners were informed about the study protocol, including the 16-day hospitalization period, potential risks, and benefits. To minimize stress, cats were housed individually in enriched enclosures with hiding boxes, elevated perches, and familiar bedding from home. Daily enrichment activities and gentle handling protocols were implemented. A quiet environment with minimal noise exposure was maintained. While this does not represent a typical home environment, standardized housing conditions were applied equally to both groups, minimizing differential stress effects on treatment comparison. This study follows reporting recommendations adapted from ARRIVE guidelines 2.0, recognizing that while these are client-owned patients rather than experimental animals, transparent reporting standards enhance reproducibility. Animals were divided into two groups:

- The study compares the effectiveness of exosomes produced from mesenchymal stem cells (MSC) with conventional wound healing methods in a double-blind, randomized controlled experiment.
- Computerized random allocation is used for randomization.
- The study is double-blinded, meaning that neither the participants nor the researchers evaluating the results are aware of the group assignment.
- Sample size was calculated using G*Power software (version 3.1.9.2) with an alpha error of 0.05, power of 80%, and an anticipated effect size of 0.377, yielding a minimum of 16 cats (8 per group). Given that this was an inpatient study with hospitalized animals and close monitoring, dropout risk was considered low. Nevertheless, to adopt a conservative approach and allow for potential unforeseen events (e.g., early humane endpoint due to unrelated health issues or protocol violations), we incorporated a 20% inflation, resulting in a planned enrollment of 20 cats. No dropouts occurred during the study.
- Control group: 10 Cats with chronic ulcer received carboxymethylcellulose (CMC) gel alone for the ulcer, with standard care.
- Extracellular vesicle (Evs) treated group: 10 Cats with chronic ulcer received CMC gel-loaded exosomes for ulcer, with standard care.

#### Inclusion criteria

Cats of any breed and gender diagnosed with non-healing skin wound or ulcer, including traumatic wounds, chronic wounds, or ulcers persisting for more than 4 weeks. Wounds must have shown no progressive healing despite at least 4–6 weeks of appropriate etiology-directed standard care. Stable medical condition apart from the wound or ulcer being treated (ASA1 classification).

#### Exclusion criteria

Age below 6 months or above 15 years old. Active systemic infection or sepsis. Presence of untreated malignancy or undergoing active cancer treatment. History of allergic reactions to stem cell-derived products or extracellular vesicles. Pregnancy or lactation in female cats. Significant coagulopathy or bleeding disorder.

#### Animal welfare and housing

- Participants are monitored over 16 days for every 4 days for adverse reactions.
- Provisions are in place for discontinuation due to medical reasons, withdrawal of consent, or protocol violations.

## Assessment and evaluation

### Clinical evaluation

#### Assessment of wound healing

The proportion of wound contraction was used to assess wound healing. Before the therapy was applied, the diameter of the wound edges decreased by mm per day for 16 days, as seen in Table 3.

#### Calculating the percent contraction of wounds

Wound contraction was measured as a percentage of the initial wound area in each animal group. The following formula was used to determine the proportion of wound contraction: percentage of closed wounds = $$\frac{wound area on day 0 - wound area on day n}{wound area on day 0}\times 100$$.

The wound diameter was measured using a meter rule, as indicated in Fig. 3 and Table 3, and n is the number of days (4, 8, 12, and 16) that we observed the non-healing ulcer wounds to evaluate our treatment approach.

The formula Area = πr^2^ (where r^2^ = diameter and π is about equal to 22/7), was used to get the wound area. The original wound area (wound area on day 0) was measured and noted. The proportion of wound contraction for each group was then calculated for each cat's wound.

#### Diagnostic work-up and management of underlying causes

Before enrollment, all cats underwent a comprehensive diagnostic evaluation to identify underlying or perpetuating factors. This included detailed history, full physical and dermatological examination, skin cytology, deep skin scrapings, flea infestation assessment, bacterial and fungal cultures (when indicated), and, where appropriate, trial elimination diets or environmental allergen minimization for suspected pruritic/allergic conditions. Cases with uncontrolled systemic disease, active malignancy, or untreated severe infection were excluded. During the study, etiology-directed treatments were continued concurrently with the trial protocol. This ensured that WJ-MSC-derived EVs were assessed as an adjunctive therapy atop optimal management of primary and perpetuating causes.

#### Preparation and topical application of treatment products

Extracellular vesicles (EVs) were resuspended in sterile phosphate-buffered saline (PBS) immediately prior to use. The protein concentration of the EV preparation was quantified using the Bradford assay, and a standardized dose of 100 μg total EV protein per cm^2^ of wound surface area was applied. EV batches confirmed a mean concentration of 2.8 × 10⁹ particles/μL (range 2.5–3.2 × 10⁹ particles/μL across batches). Thus, the applied dose corresponded to approximately 2.8 × 10^11^ EV particles per cm^2^ of wound area. Carboxymethyl cellulose (CMC) gel (2% w/v, sterile) was used as the vehicle. For the EV-treated group, the required volume of concentrated EV suspension was homogeneously mixed into the CMC gel immediately before application to achieve the target protein dose based on the measured initial wound area. For the control group, an equivalent volume of PBS was incorporated into the CMC gel. A uniform layer of gel approximately 2 mm thick was applied, corresponding to approximately 0.2 mL of gel per cm^2^ of wound surface area. The treated area included the wound bed and a 5 mm perimeter of perilesional skin. Standard care, provided identically to both groups, included etiology-directed systemic or topical medications as clinically indicated: antibiotics (amoxicillin-clavulanate or cefovecin based on culture and sensitivity results in infected cases), antipruritic therapy (oclacitinib or cyclosporine for pruritus-related cases), and analgesic management (buprenorphine or meloxicam as needed). Wound cleaning with sterile saline and protective bandaging were performed daily in all cats.

#### Pain assessment

Pain associated with the chronic ulcers was assessed as a secondary outcome using the validated Feline Grimace Scale (FGS), a reliable and well-established tool for evaluating acute and chronic pain in cats. Scoring was performed daily by a single trained observer blinded to treatment allocation. The FGS evaluates five action units (ear position, orbital tightening, muzzle tension, whiskers position, and head position), each scored from 0 (absent) to 2 (markedly present), yielding a total score out of 10. Scores ≥ 4/10 are considered indicative of pain requiring analgesic intervention.

### Histopathological assessment

On the third, seventh, fourteenth, and twenty-first days after treatment, skin samples from the lesion site were collected and prepared for histological assessment using hematoxylin and eosin (H&E) staining. Furthermore, full-thickness 6-mm punch biopsies were taken from the wound margin, including both lesional and adjacent healthy tissue to allow comparison of healing progression. A single biopsy was collected per time point per animal. Biopsy sites were closed with non-absorbable sutures and managed with standard post-procedure care. To minimize animal burden, biopsies were staggered such that no cat received more than two punches over the study period (e.g., Days 3 and 14, or Days 7 and 21).Photomicrographs were captured, and the histological architecture of the wounds was compared at these four time points. To evaluate the healing of the wound surface, histopathological results were assessed using a scoring system based on the morphology of the epidermis, the distance between the wound edges, and the direction of the collagen fibers and fibroblasts as shown in Tables 1 and 2 and Figures from 6, 7, 8 and 9.

**Table 1:** Mean wound area (cm^2^) and percentage of wound reduction in EV-treated and control groups over multiple observation points (T0 = admission, T4 = day 4, T8 = day 8, T12 = day 12, T16 = day 16)

Time point	Wound area (cm^2^) EV-treated group (*n* = 10)	Wound area (cm^2^) Control group (*n* = 10)	Percentage wound reduction (%) EV-treated group	Percentage wound reduction (%) Control group	Mean difference in % reduction (95% CI)
T0 (admission)	5.9 ± 2.4 (95% CI 4.2–7.6)	6.1 ± 2.2 (95% CI 4.5–7.7)	0.0 ± 0.0 (95% CI –)	0.0 ± 0.0 (95% CI –)	–
T4 (day 4)	4.8 ± 2.0 (95% CI 3.4–6.2)	5.4 ± 2.1 (95% CI 3.9–6.9)	18.6 ± 7.9 (95% CI 13.0–24.2)	11.5 ± 6.8 (95% CI 6.6–16.4)	7.1% (95% CI 0.4–13.8%)
T8 (day 8)	3.1 ± 1.5 (95% CI 2.0–4.2)	4.6 ± 1.8 (95% CI 3.3–5.9)	47.5 ± 11.2 (95% CI 39.5–55.5)	24.6 ± 9.8 (95% CI 17.6–31.6)	22.9% (95% CI 13.7–32.1%)*
T12 (day 12)	1.4 ± 0.9 (95% CI 0.8–2.0)	3.3 ± 1.4 (95% CI 2.3–4.3)	76.3 ± 9.5 (95% CI 69.5–83.1)	45.9 ± 12.3 (95% CI 37.1–54.7)	30.4% (95% CI 21.0–39.8%)**
T16 (day 16)	0.4 ± 0.3 (95% CI 0.2–0.6)	2.5 ± 1.0 (95% CI 1.8–3.2)	92.4 ± 6.8 (95% CI 87.5–97.3)	58.3 ± 12.1 (95% CI 49.6–67.0)	34.1% (95% CI 25.6–42.6%)***

Data show faster wound contraction in the EV group, indicating superior healing efficacy. Mean between-group difference at Day 16 with 95% confidence interval: 34.1% (25.6% to 42.6%) for percentage wound reduction

Data are presented as mean ± SD (95% CI)

Percentage wound reduction calculated relative to T0 for each cat

*P < 0.05, **P < 0.01, ***P < 0.001 (Student’s t-test, EV-treated vs. control at each time point)

**Table 2:** Baseline characteristics and wound etiologies of enrolled cats by treatment group using EVs, including ulcer score, surface area, and healing time, highlighting treatment variability among cases and the overall effectiveness of EV-based therapy

Parameter	EV-treated group (n = 10)	Control group (n = 10)	p-value
Age (years)	7.2 ± 2.9 (range: 3–12)	7.5 ± 3.0 (range: 3–13)	0.80
Sex			> 0.99
Male	5 (50%)	5 (50%)	
Female	5 (50%)	5 (50%)	
Body weight (kg)	4.7 ± 1.1 (range: 3.1–7.2)	4.9 ± 1.2 (range: 3.3–7.0)	0.72
Wound duration (weeks)	9.4 ± 2.5 (range: 6.5–14)	9.1 ± 2.3 (range: 7–13)	0.76
Initial wound area (cm^2^)	5.9 ± 2.4 (range: 2.1–10.4)	6.1 ± 2.2 (range: 2.5–10.0)	0.81
Ulcer score (0–10)	6.5 ± 1.6 (range: 4–9)	6.7 ± 1.5 (range: 4–9)	0.78
Wound etiology			P > 0.99*
Traumatic (bite/laceration)	4 (40%)	3 (30%)	
Post-surgical dehiscence	2 (20%)	3 (30%)	
Pressure sore	2 (20%)	2 (20%)	
Pruritus-related self-trauma (allergic/immune-mediated)	2 (20%)	2 (20%)	

#### Biopsy Sampling and Rationale

Skin biopsies were collected under sedation or light anesthesia on Days 3, 7, 14, and 21 post-treatment initiation to assess the temporal progression of histological healing and tissue remodeling. Sampling on Days 3 and 7 captured early inflammatory and proliferative phases, Day 14 represented mid-stage granulation and re-epithelialization, and Day 21 allowed evaluation of late-stage collagen maturation and epidermal restoration after cessation of topical treatment. Clinical wound monitoring and treatment application concluded at Day 16 for all animals. However, to obtain the Day 21 histological endpoint without extending daily interventions, treatment was discontinued at Day 16, and animals were housed until biopsy on Day 21. All cats were returned to owners or adopted after final sampling.

### Immunohistochemical (IHC) for assessment of wound vascularization

Immunohistochemistry for α-SMA and CD31 were performed on serial sections from the same formalin-fixed, paraffin-embedded biopsy samples used for H&E staining. Thus, only one biopsy was required per time point. On days 3, 7, 14, and 21, immunohistochemistry (IHC) was carried out. Primary antibodies (anti-CD31, 1:100, Abcam, United Kingdom) were incubated with the tissue sections for an entire night at 4°C. HRP-coupled secondary antibodies (Aspen, China) were then added. The staining process was carried out with diaminobenzidine (DAB). Tube-like structures were counted for each area and recognized as newly created blood vessels. Furthermore, alpha-smooth muscle actin (α-SMA) DAB-peroxidase staining may indicate persistent fibrotic processes, which may be linked to persistent inflammation or scarring. Effective wound healing and tissue repair depend on active angiogenesis, which is indicated by CD31, which is a positive CD31 staining.

## Outcome measures

- Primary Outcome: Percentage reduction in wound size in 16 days.
- Secondary Outcomes: Granulation tissue formation, epithelialization rates, and pain assessment via investigator-reported outcomes.

## Data collection and reporting

**Fig. 4:**
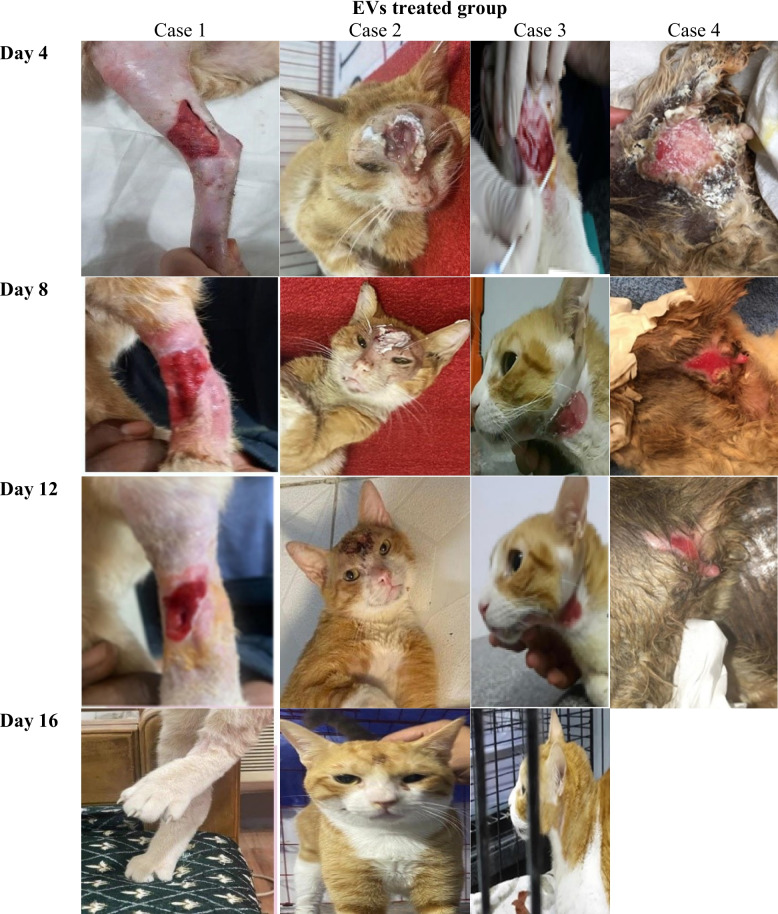
Illustrates the progression of wound healing in the extracellular vesicle (EV)-treated group compared to the control group over 16 days. Serial images captured on days 4, 8, 12, and 16 shows a clear distinction in wound closure rates in the EV-treated group

- Wound size measured using a meter rule as shown in Fig. 4 and Table 3.
- Granulation tissue, infection markers, and pain levels were documented during the visits, over the 4 days.

**Table 3:** Scoring system for wound surface healing assessment based on epidermis morphology and wound edge distance

Score	Morphology of the epidermis	Distance between wound edges
0—Near normal	Completely regenerated	< 500 µm
1- Highly mature	> 80% regenerated	< 1000 µm
2—Mature	> 50% regenerated	< 1500 µm
3 – Immature	< 20% regenerated	< 2000 µm
4 – Highly immature	No epidermis formed	> 2000 µm

Scores range from 0 (near normal) to 4 (highly immature), reflecting varying degrees of regeneration and healing completeness.

### Statistical analysis

In this study, data were analyzed using SPSS 18.0 software. Group comparisons at individual time points were performed using Student's t-test, with significance levels: *P < 0.01 and **P < 0.001. Results are expressed as mean ± standard deviation (SD). To improve interpretability of the primary outcome as suggested during review, 95% confidence intervals (95% CI) for the mean between-group differences in wound area and percentage wound contraction are also reported in the Results section and relevant Figures/Tables.

## Results

### Stem cells characterization

The morphological appearance of WJ-MSCs was observed under an inverted microscope, cells typically exhibit a spindle-shaped, large and flattened morphology with a pronounced fibroblastic appearance displaying prominent cytoplasmic extensions. Their uniform size and elongated shape indicate healthy proliferative potential, as it was shown in Fig. (1A) with a confluence of 70% and in Fig. (1B) showing a complete sheet.

Flow cytometry showed high expression of mesenchymal markers CD90 (99%) and CD73 (91.2%), with low levels of CD34 (0.88%) and CD45 (0.29%), which is characteristic of mesenchymal stem cells; these are clearly shown in Fig. 1 (C-F).

### Wound healing or scratch assay analysis and WJ-MSC EVs characterization

The scratch assay results, illustrated in Fig. 3(A) and (B), demonstrate the effect of Wharton Jelly Mesenchymal Stem Cell (WJ-MSC)-derived extracellular vesicles (EVs) on cell migration. The migration rate, expressed as the percentage of scratch closure, was significantly higher in the EV-treated group compared to the control group at 0, 12, 24, and 48 h.

In the control group, cell migration rates were 5.0 ± 2.5% (95% CI 3.2–6.8%) at 0 h, reflecting initial measurement variation and minor spontaneous cell movement immediately after scratch creation, rather than active migration. This baseline difference was not statistically significant, increasing gradually to 15.0 ± 4.2% (95% CI 12.0–18.0%) at 12 h, 20.0 ± 5.1% (95% CI 16.4–23.6%) at 24 h, and 35.0 ± 8.9% (95% CI 28.6–41.4%) at 48 h. In the WJ-MSC EV-treated group, migration rates were 10.0 ± 3.6% (95% CI 7.4–12.6%) at 0 h, rising to 30.0 ± 6.3% (95% CI 25.5–34.5%) at 12 h, 40.0 ± 7.8% (95% CI 34.4–45.6%) at 24 h, and 60.0 ± 7.2% (95% CI 54.8–65.2%) at 48 h. At 24 h, the control group showed a migration rate of 20.0 ± 5.1% (95% CI 16.4–23.6%), while the WJ-MSC EV-treated group exhibited 40.0 ± 7.8% (95% CI 34.4–45.6%); mean difference 20.0% (95% CI 13.7–26.3%, *P < 0.01).These results indicate that WJ-MSC-derived EVs enhance cell migration effectively, facilitating faster scratch closure.

The isolated extracellular vesicles (EVs) from Wharton’s Jelly mesenchymal stem cells were characterized by flow cytometry and transmission electron microscopy (TEM). As shown in Fig. 2 (A, B, C), flow cytometric analysis revealed that the vesicles expressed the extracellular vesicle surface markers CD9, CD63, and CD81. The positive populations for CD9, CD63, and CD81 were 88.7%, 91.0%, and 89.2%, respectively, indicating high purity and successful isolation of exosomes. Furthermore, TEM analysis (Fig. 2D) demonstrated that the vesicles exhibited the characteristic round, cup-shaped morphology, with a mean diameter of 34.7 ± 5.2 nm (95% CI 32.1–37.3 nm).The vesicles appeared as spherical, membrane-bound nanostructures with a clear lipid bilayer, consistent with the known morphology of mesenchymal stem cell-derived exosomes.

### Measurement of wound area

All 20 enrolled cats (supplementary file) completed the 16-day study protocol with no dropouts or protocol deviations. Figure 4 illustrates the progression of wound healing in the extracellular vesicle (EV)-treated group compared to the control group over a 16-day period. Serial images captured on days 4, 8, 12, and 16 shows a clear distinction in wound closure rates in the EV-treated group. By Day 4, the EV-treated group displayed noticeable wound contraction. On Day 8, the EV-treated wounds exhibited further reduction in wound size, with granulation tissue visibly forming, which is essential for tissue repair. By Day 12, the EV-treated wounds demonstrated advanced healing, with substantial wound closure and a more organized appearance of the wound bed. Finally, by day 16, the EV-treated group exhibited near-complete wound closure with a mean wound contraction of 92.4 ± 6.8% compared to 58.3 ± 12.1% in the control group (mean difference 34.1%, 95% CI 25.6% to 42.6%). Residual wound area was correspondingly reduced in the EV-treated group (mean difference –3.8 cm2, 95% CI –5.2 to –2.4 cm2).

**Fig. 5:**
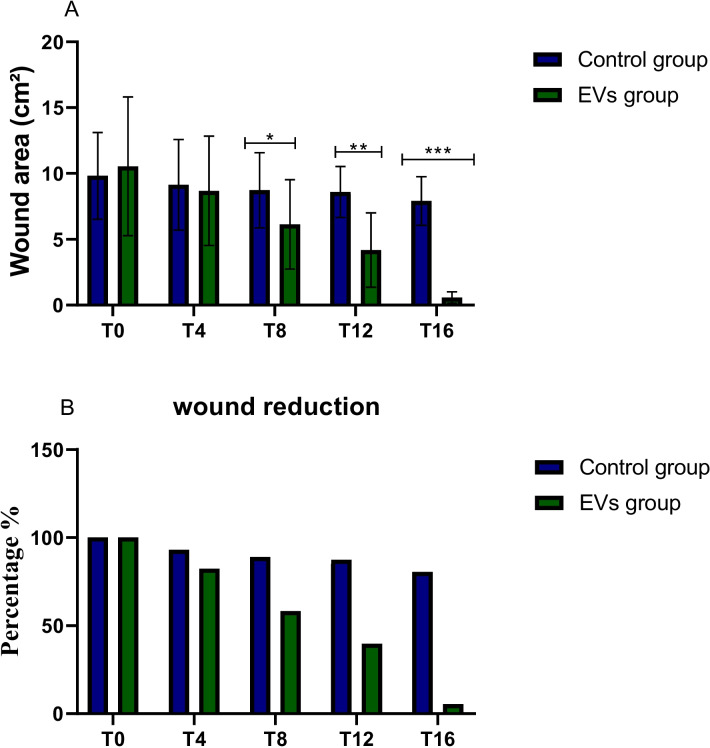
Comparison of wound healing progression between groups. **A** Wound area (cm^2^) and **B** percentage wound reduction over time. Values are mean ± SD (n = 10 per group). Mean between-group differences at Day 16 with 95% CI are provided in the text (Sect. "Measurement of Wound Area"). Significance markers: dii < 0.05, **P < 0.01, ***P < 0.001 (Student’s t-test)

Figure 5 and Table 1 demonstrates that the EV-treated group exhibited significantly accelerated wound healing compared to the control group. By T8, T12, and T16, the EV group showed a marked reduction in wound size and a higher percentage of wound closure, achieving near-complete closure at T16, while the control group displayed slower healing progress (*P < 0.05, **P < 0.01, ***P < 0.001).

Table 2 Baseline demographic and wound characteristics were comparable between the EV-treated and control groups, confirming successful randomization and absence of major confounding variables at enrollment.

Data are presented as mean ± standard deviation or n (%). *p*-values calculated using Student’s t-test for continuous variables and Fisher’s exact test for categorical variables (etiology distribution). *No significant differences were observed between groups (all p > 0.05), confirming baseline comparability.

### Histopathological results

**Fig. 6:**
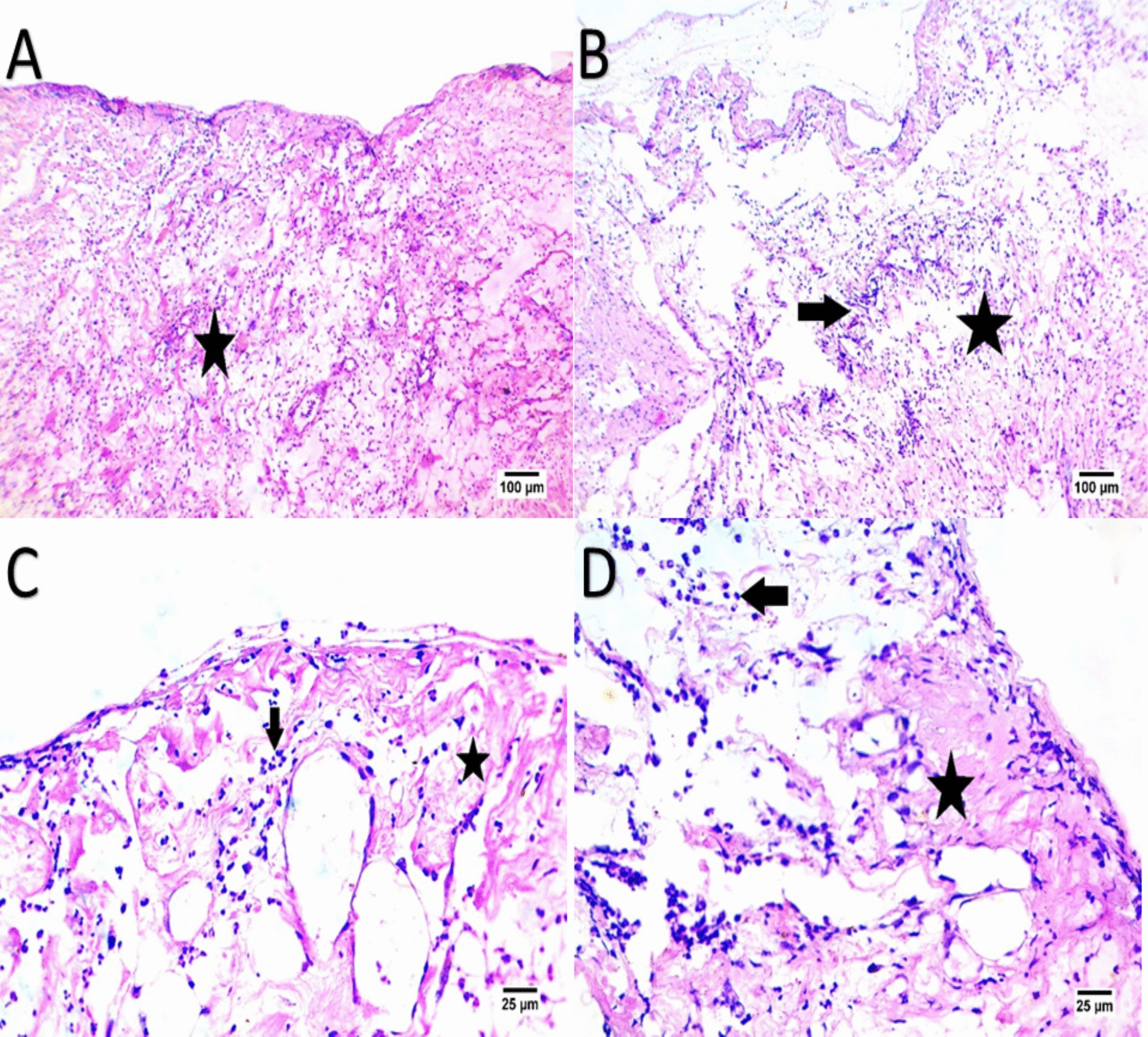
Panels (**A**, **B**, **C**, **D**) depict H&E-stained wound tissue on Day 3, comparing EV-treated (**A**, **C**) and control groups (**B**, **D**). The EV-treated group shows initial formation of fibrous connective tissue (indicated by the star) with moderate mononuclear inflammatory cell infiltration (indicated by the arrow), indicating early healing. In the control group (**B**, **D**), connective tissue appears disorganized, with a higher density of inflammatory cells, suggesting a delayed healing response

**Fig. 7:**
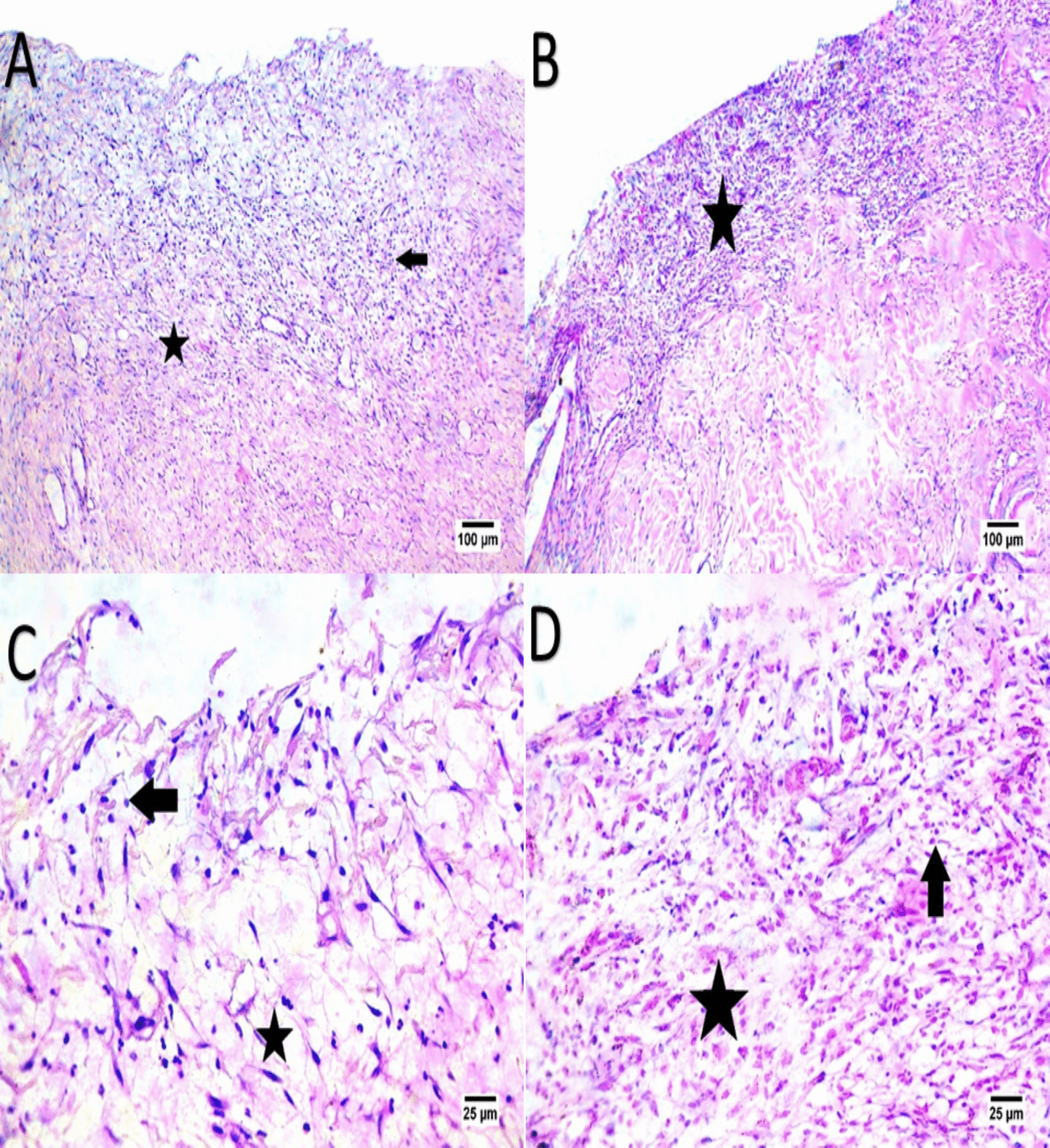
Panels (**A**, **B**, **C**, **D**) display Day 7 histology in H&E-stained sections. The EV-treated (**A**, **C**) wounds show better-organized fibrous tissue (indicated by the star) and fewer inflammatory cells (indicated by the arrow), suggesting continued tissue maturation. In the control group (**B**, **D**), connective tissue remains less organized with sustained inflammatory cell presence, indicating a slower healing process

**Fig. 8:**
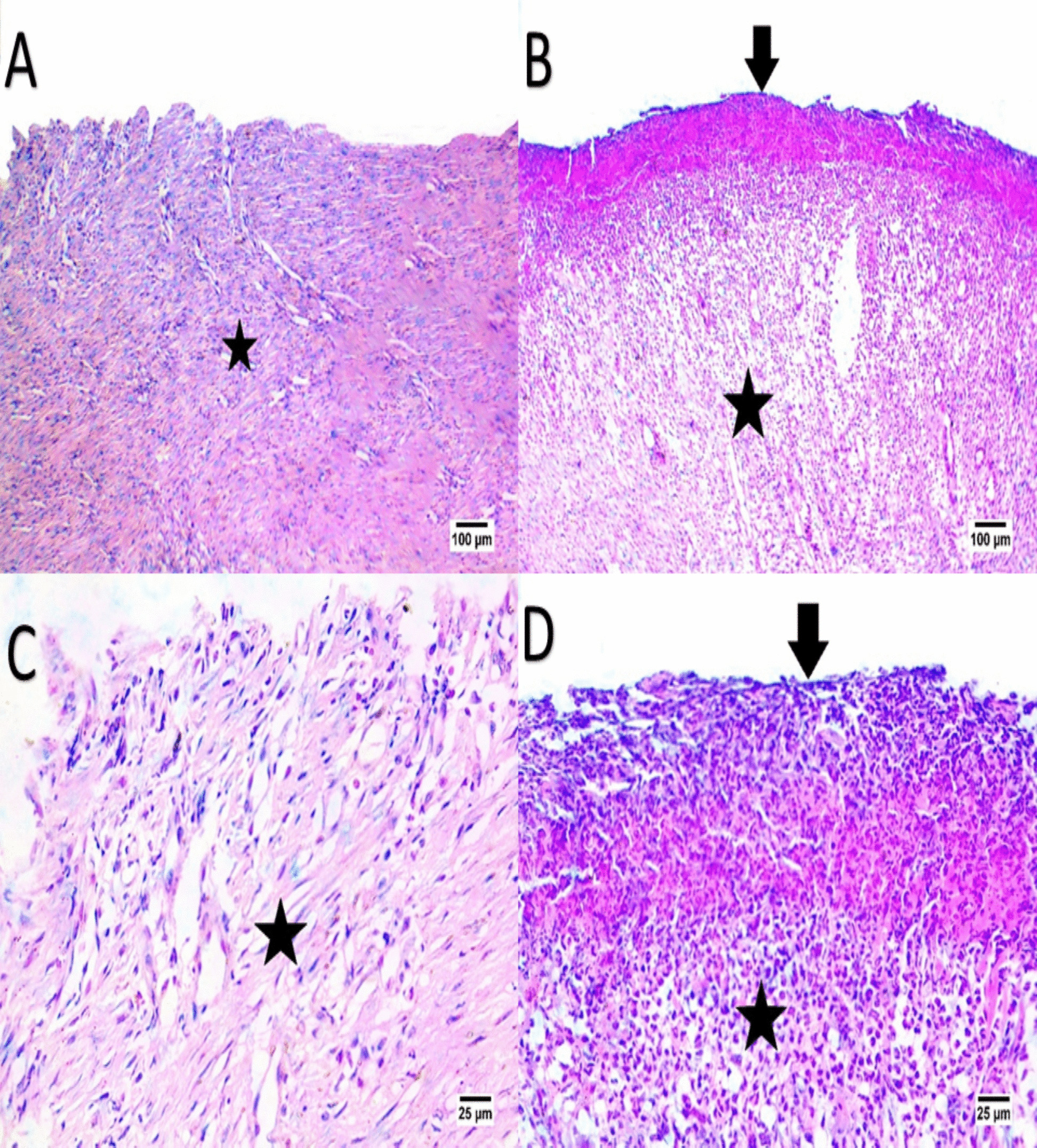
Panels (**A**, **B**, **C**, **D**) represent Day 14 histological findings. EV-treated (**A**, **C**) samples exhibit well-organized connective tissue (indicated by the arrow) with minimal inflammatory infiltration (indicated by the star) and partial epidermis formation, indicating advanced healing. The control group (**B**, **D**) shows moderate organization and persistent inflammation, suggesting a slower recovery

**Fig. 9:**
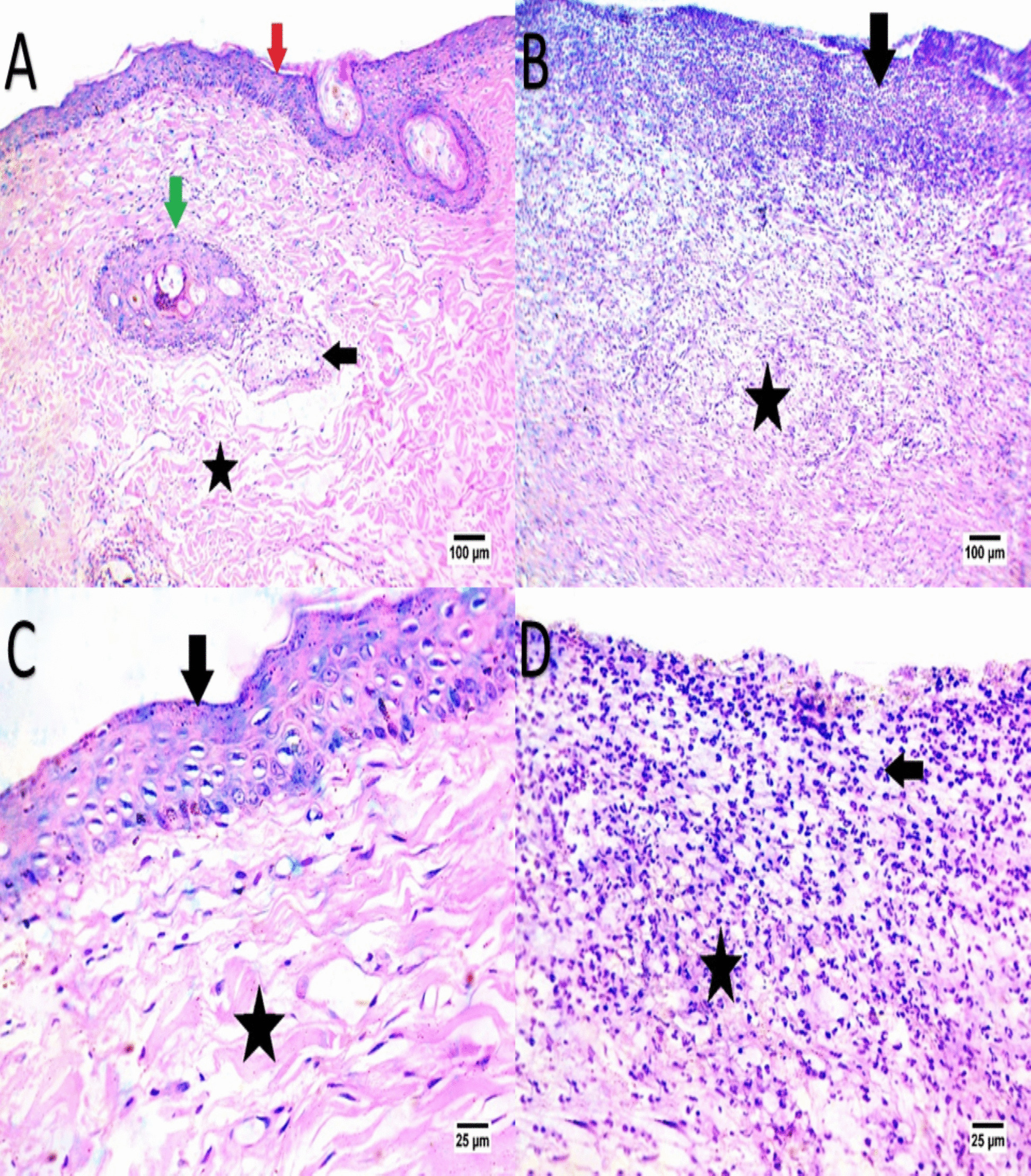
Panels (**A**, **B**, **C**, **D**) illustrate Day 21 results, with EV-treated (**A**, **C**) wounds showing mature, organized connective tissue, complete epidermal layer formation (indicated by star), and minimal inflammation (indicated by arrow). The control group (**B**, **D**) shows incomplete tissue organization (indicated by star) with remaining inflammatory cells (indicated by arrows), highlighting the enhanced healing effect of EV treatment

The wound sections stained with hematoxylin and eosin (H. E) are shown in Figs. 6, 7, 8 and 9 as regard EVs group the results showed healing was characterized by the formation of well-organized fibrous connective tissue and inflammatory cells decreased with formation of epidermis and sweat gland, enhanced cellularity and increased vascularization, also increased keratinocytes with complete re-epithelialization of wound site, however, there were many undifferentiated epidermal cells. The dermis at the wound site, showing enhanced dermal fibroblasts and collagen fiber deposition, however, as regards CMC group healing, was characterized by formation of fibrous connective tissue accompanied by a high infiltration of mononuclear inflammatory cells and covered by scab, is shown in Figs. 6, 7, 8 and 9, Table 3, and Table 4.

**Table 4:** Evaluation criteria for wound healing maturity using collagen and fibroblast alignment

Score	Direction of collagen fibers	Direction of fibroblast
0—Near normal	Almost all in one direction	Parallel collagen fibers
1- Highly mature	Mostly in one longitudinal direction, but with few areas of unorganized collagen fibers	> 75% in the direction of collagen fibers
2 – Moderately Mature	More than one longitudinal direction	> 50% in the direction of collagen fibers
3 – Immature	Irregular orientation, collagen fibers have some orientation, but orientation is not longitudinal (e.g. circular pattern)	> 25% in the direction of collagen fibers
4 – Highly Immature	No diagnostic orientation	No orientation

Scores from 0 to 4 represent collagen orientation, with lower scores indicating better-organized, healing tissue architecture

### Assessment of wound vascularization using IHC

**Fig. 10:**
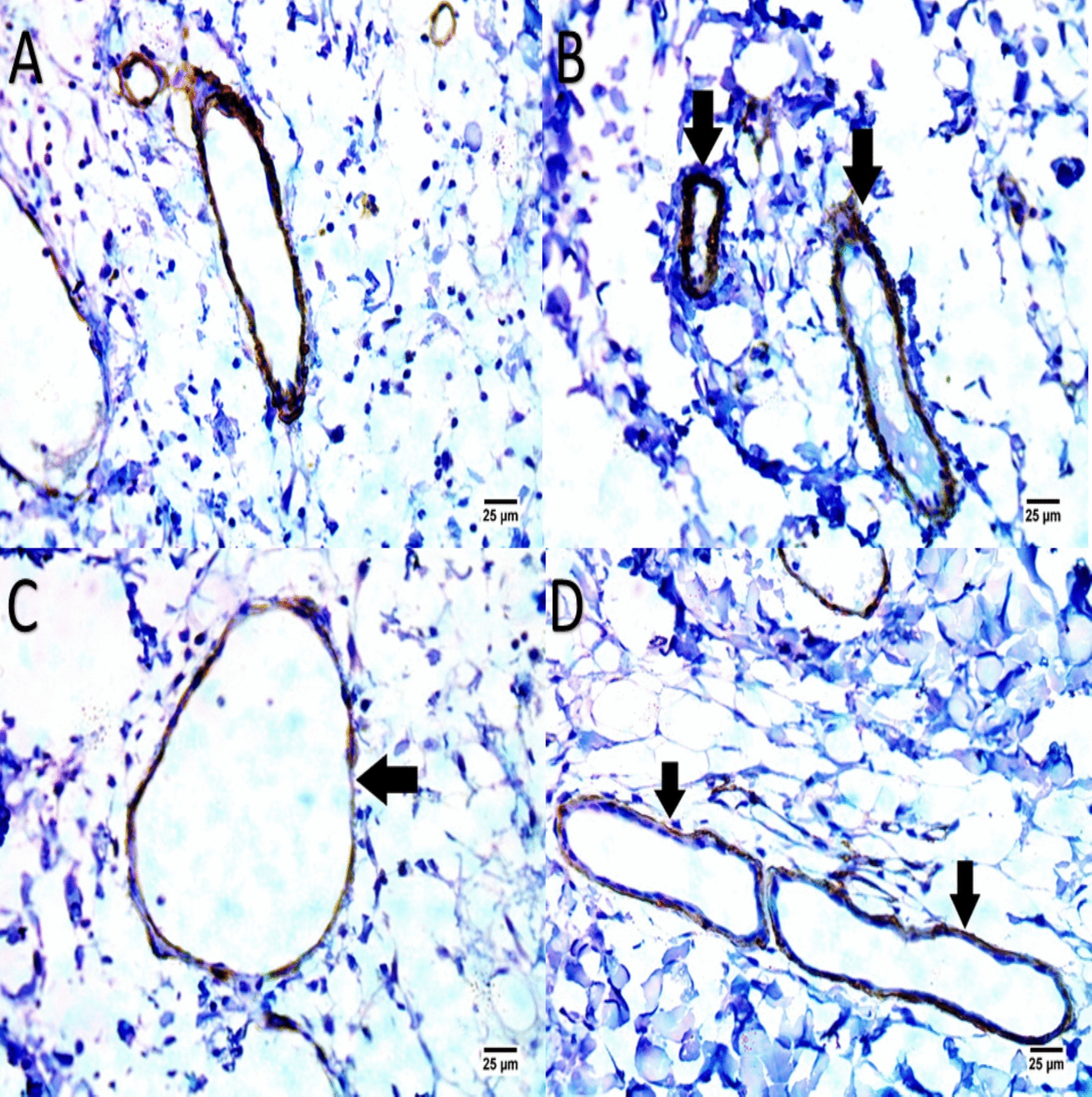
Panels (**A**, **B**, **C**, **D**) present IHC-stained images for α-SMA and CD31 on Day 3, highlighting vascularization in EV-treated versus control groups. The EV-treated group shows positive α-SMA and CD31 staining (**A**, **C**), indicating new blood vessel formation, while the control group exhibits less vascular activity (**B**, **D**), marking a slower angiogenic response

**Fig. 11:**
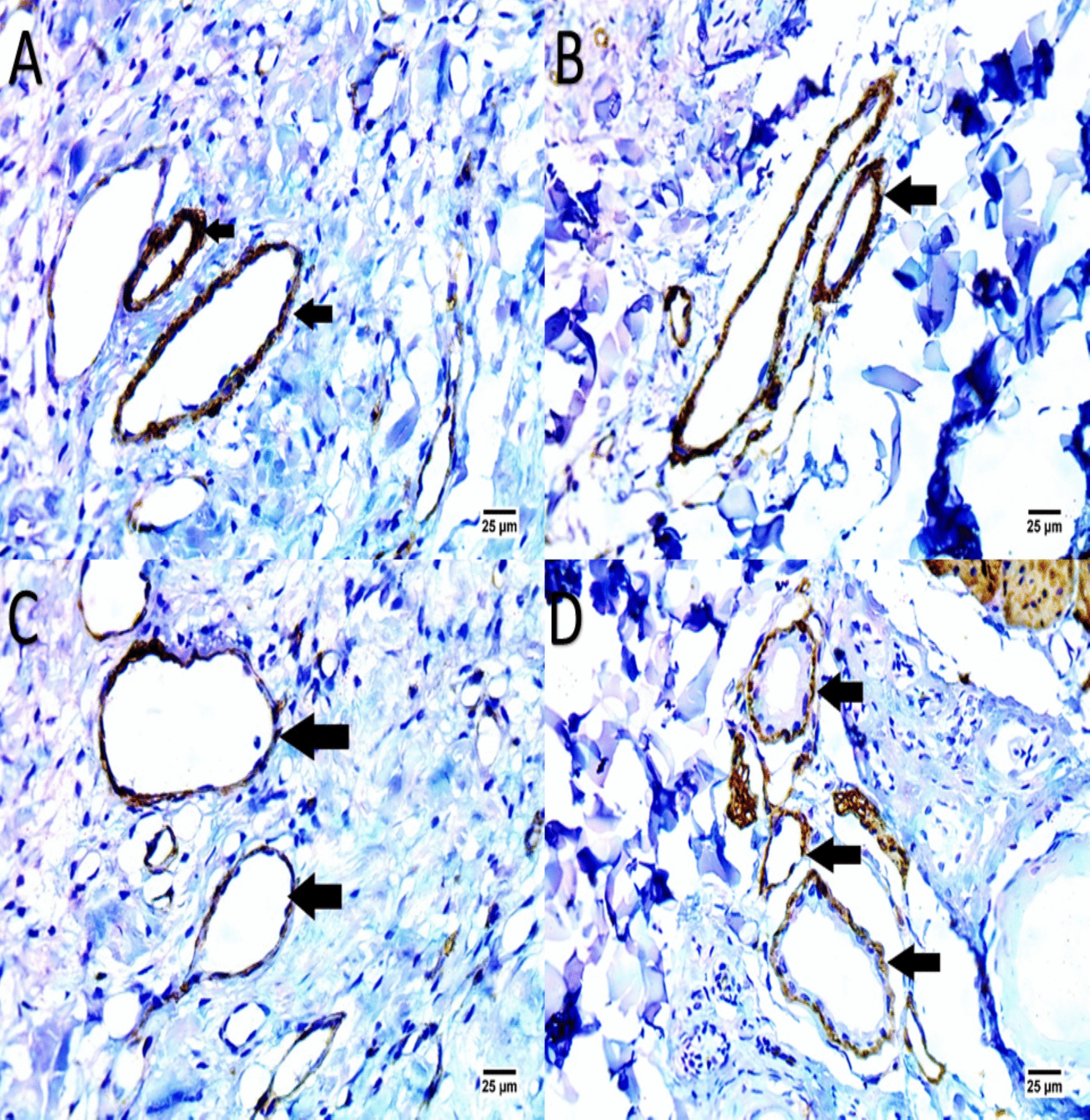
Panels (**A**, **B**, **C**, **D**) show IHC staining on Day 7. EV-treated wounds display strong α-SMA and CD31 staining (**A**, **C**), suggesting robust angiogenesis and enhanced vascular support for tissue repair. In contrast, the control group has weaker staining (**B**, **D**), indicating slower vascular development and delayed healing

Immunohistochemical analysis using α-SMA and CD31 staining revealed significant differences in angiogenesis between the EV-treated and control groups (Fig. 10, 11). In the EV-treated wounds, α-SMA staining demonstrated a positive reaction in the walls of newly formed blood vessels, indicating active fibroproliferative processes and tissue repair. CD31 staining was also prominently positive in these samples, reflecting robust angiogenesis, a critical component of effective wound healing. This enhanced vascularization was observed as early as days 3 and 7 post-treatment in the EV-treated group, suggesting that extracellular vesicle treatment promotes an accelerated vascular response compared to the control group. Conversely, the control group exhibited minimal α-SMA and CD31 expression, indicating limited new vessel formation and a slower healing progression.

**Fig. 12:**
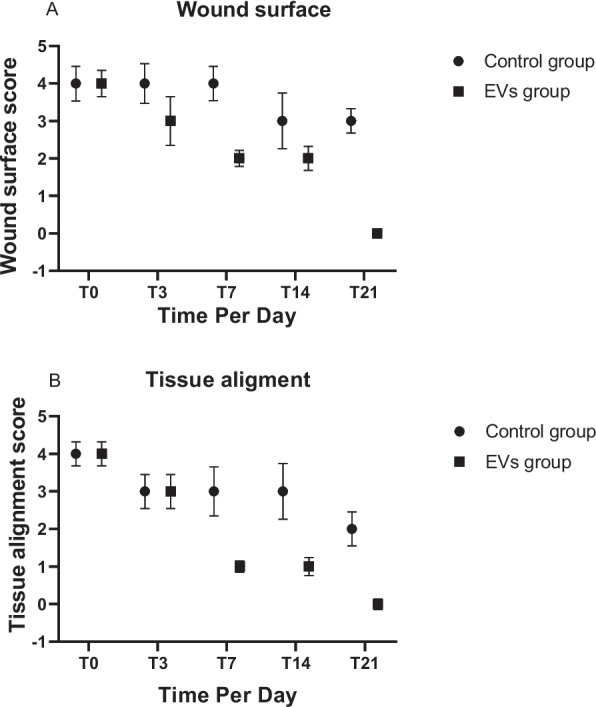
illustrates the comparison between the control group and the extracellular vesicle (EVs) group across different time points (T0, T3, T7, T14, T21). **A** represents the wound surface assessment score, where lower scores indicate better healing. At T0, both groups had the same score (4), indicating similar baseline wound conditions. Over time, the EVs group showed a significantly faster reduction in scores, reaching complete healing score (0) by T21, while the control group remained at a score of (3). **B** represents tissue alignment scores, where lower scores indicate better tissue organization. At T0, both groups started at the same score (4). The EVs group demonstrated superior tissue organization as early as T7 score (1) and achieved perfect alignment score (0) by T21, while the control group remained at a score of (2)

Figure 12 highlights the superior wound healing and tissue organization achieved with EV treatment compared to the control group. By T21, the EV group reached complete healing and perfect tissue alignment (score = 0), while the control group showed slower progress, with residual wound surface (score = 3) and suboptimal tissue organization (score = 2).

### Secondary outcomes

Pain scores, assessed using the Feline Grimace Scale, were low at baseline in both groups (EV-treated: 3.2 ± 1.1; Control: 3.4 ± 1.0; p = 0.71) and showed gradual reduction over the study period, consistent with progressive wound healing. No significant differences were observed between groups at any time point (p > 0.05). No cat required additional rescue analgesia beyond the standard pain management protocol provided as part of etiology-directed care.

## Discussion

The results of this randomized controlled trial demonstrate that topical WJ-MSC-derived EVs significantly accelerate healing in naturally occurring chronic skin ulcers in cats, addressing a common and therapeutically challenging condition in veterinary dermatology [34–36]. Until recently, chronic wound care often relied on outdated practices. Today, however, advancements in prevention, diagnosis, and treatment have revolutionized care for these persistent conditions. This progress stems from deeper insights into cellular and molecular biology, cutting-edge biomedical engineering innovations, and enhanced capacity for rigorous, reliable clinical research [27, 37, 38].

The findings from the present study align with Chen et al. showed that by triggering the ERK/MAPK pathway, exosomes generated from ADSCs may increase the ratios of collagen I to collagen III, TGF-β3 to TGF-β1, and MMP3 to TIMP1. This activation aid in preventing the differentiation of fibroblasts into myofibroblasts [30, 39]. Through the upregulated expression of CD31 and α-SMA, which highlights the active growth of endothelial and smooth muscle cells as seen in our study, MSC exosomes not only accelerate re-epithelialization but also stimulates the development of important skin structures, such as sebaceous glands and hair follicles, signaling robust vascularization necessary for tissue regeneration [40].

The enhanced cell migration observed in the in vitro scratch assay supports the pro-migratory effects of WJ-MSC-derived EVs, likely contributing to the accelerated re-epithelialization and wound contraction seen clinically in the feline ulcers. This suggests that WJ-MSC-derived EVs play a crucial role in promoting cell migration and accelerating wound healing processes [35, 41]. Clinically, the EV-treated group's wound size significantly shrank by the fourth day after treatment, and by the sixteenth day, it had almost fully healed, significantly outperforming the control group. This beneficial effect is probably caused by EVs' capacity to control the inflammatory response in the early phases of healing, especially by triggering macrophage migration. M2c macrophages are distinguished by their high IL-10, MMP-9, IL-1β, and TGF-β production while maintaining low IL-12 levels. Additionally, they express CD163, a hemoglobin receptor that is essential for anti-inflammatory reactions [42].

The findings from the present study align with previous studies showing that EVs derived from human umbilical cord mesenchymal stem cells (huc-MSCs) have been shown by Li et al. to control macrophage activity in burned rats through miR-181C, inhibiting the TLR4 signaling pathway and lowering IL-10 levels, which encourage M2 polarization and control the inflammatory response [43–45]. In the present study, the absence of significant inter-group differences in pain scores may reflect the relatively mild baseline discomfort associated with these chronic ulcers, as well as effective underlying pain management in both groups. The relatively small sample size (n = 10 per group) limits statistical power and generalizability. Although baseline characteristics and wound etiologies were balanced between groups, larger multicenter studies are needed to confirm efficacy across diverse feline populations, breeds, and clinical settings.

In a dose-dependent manner, EVs made from adipose stem cells (ASCs) accelerate cutaneous fibroblast proliferation, report Kim et al. [46]. Additionally, Gangadaran et al. found that EVs released from huc-MSCs stimulate skin cells' β-catenin signaling through the Wnt4 protein, improving their migratory and proliferative capabilities and reversing the blockage of the AKT pathway to increase skin cell survival [47, 48].

The marked increase in CD31-positive vessel density and α-SMA expression observed in the EV-treated group as early as Days 3 and 7 indicates that WJ-MSC-derived EVs induce rapid and robust angiogenesis, which appears to be a primary driver of the accelerated wound closure seen in this feline chronic ulcer model [36, 49, 50]. This early vascular response likely improved oxygen and nutrient delivery to the wound bed, facilitated recruitment of reparative cells, and supported subsequent re-epithelialization and collagen maturation processes that are frequently delayed or dysregulated in feline non-healing ulcers due to persistent inflammation, self-trauma, or inadequate granulation tissue formation [41, 51]. The temporal pattern is particularly informative: angiogenesis preceded the major acceleration in wound contraction (evident from Day 8 onward) and the histological evidence of mature tissue remodeling by Day 21. This sequence suggests a mechanistic cascade in which EV-induced endothelial activation and myofibroblast differentiation create a more permissive environment for later proliferative and remodeling phases. While we cannot exclude contributory effects from anti-inflammatory actions or direct stimulation of keratinocyte/fibroblast migration (as seen in the scratch assay), the consistent early vascular enhancement in the absence of similar changes in controls strongly implicates angiogenesis as a dominant mechanism in this context. From a clinical perspective, the ability to stimulate early and effective neovascularization is especially relevant for managing chronic ulcers in cats, where poor vascular supply often perpetuates the non-healing state even after control of infection or pruritus [52]. By addressing this critical bottleneck, EV therapy has the potential to shorten treatment duration, reduce the need for prolonged bandaging or systemic drugs, and improve long-term tissue quality outcomes that would meaningfully decrease patient discomfort and owner burden in everyday veterinary practice [53].

This study comes with a few important limitations. Additionally, using just one animal model limits how well the results might translate to other species, including humans. The study concentrated on short-term healing results over 16 days, leaving long-term outcomes like tissue durability and potential scarring unexplored. Although extracellular vesicles (EVs) were tested against a control group, their effectiveness was not compared to other advanced wound-healing treatments, limiting the broader context of their efficacy. The study didn’t investigate how differences in EV composition shaped by things like donor tissue or the methods used to isolate them might affect the results, which raises questions about how consistent the findings would be in other scenarios. On top of that, while it did measure cell growth and blood vessel formation, diving deeper into the immune system’s role and healing pathways could have revealed more about how these effects work. Also, several cats lacked complete medical histories as they were strays or recently rescued animals at enrollment. This limited our ability to fully characterize pre-treatment duration and prior therapeutic interventions, potentially affecting baseline wound characteristics. A major limitation is the extreme etiological heterogeneity of included ulcers (trauma, dehiscence, pressure ulcers, allergic dermatitis, immune-mediated conditions). With only 10 cats per group, stratification by etiology was not feasible. Different underlying causes may respond differently to EV therapy, limiting the generalizability of our findings. Larger studies with etiology-specific subgroup analyses are needed. While Cats received different systemic treatments (various antibiotics, antipruritic agents, analgesics) based on clinical indication, introducing therapeutic heterogeneity as a potential confounding factor. While standard care was provided equally to both groups, differential effects of concurrent medications on wound healing cannot be excluded. Also, multiple punch biopsies (up to two per cat) were performed in relatively small wounds throughout the study period. These procedures, while necessary for histological assessment, may have interfered with the natural wound healing process through mechanical disruption and local inflammation. This could potentially affect the magnitude of treatment effects observed, particularly in smaller wounds.

## Conclusion

This study demonstrates that topical application of Wharton’s Jelly mesenchymal stem cell-derived extracellular vesicles (WJ-MSC-EVs), delivered in carboxymethyl cellulose gel, significantly accelerates healing of chronic skin ulcers in cats that are refractory to standard care. The treatment promoted faster wound contraction, enhanced re-epithelialization, improved collagen organization, reduced inflammation, and stimulated early angiogenesis all key deficits commonly observed in feline non-healing ulcers caused by persistent pruritus, self-trauma, recurrent infections, or traumatic/post-surgical complications. From a practical standpoint, WJ-MSC-EVs offer several advantages for daily veterinary clinical use: they provide a cell-free, off-the-shelf regenerative therapy with low immunogenicity risk, simple topical application, and no requirement for donor harvesting or complex cell culture. In feline patients, this approach has the potential to shorten treatment duration, reduce the need for prolonged systemic medications (e.g., long-term antibiotics or corticosteroids), decrease owner burden through fewer clinic visits, and improve quality of life by minimizing discomfort and scarring. Given the high prevalence of refractory chronic ulcers in cats and the limitations of current therapies, these findings support the introduction of EV-based adjunctive treatment as a promising, feasible option in veterinary dermatology and wound management. Future clinical studies in larger cohorts and multicenter settings will be valuable to confirm efficacy across different feline breeds, wound etiologies, and practice settings, and to optimize dosing and delivery protocols.

## Supplementary Information

Supplementary Material 1.

## Data Availability

No datasets were generated or analysed during the current study.
