# Supplementary material for: Healing the unhealable: Wharton's jelly stem cell vesicles as a breakthrough for feline chronic skin ulcers
Source: Ir Vet J. 2026 Apr 14;79:24. doi: 10.1186/s13620-026-00341-7 (PMC13077950; doi:10.1186/s13620-026-00341-7)
Supplement: Supplementary file 1 — Supplementary Material 1. [file 13620_2026_341_MOESM1_ESM.docx]

**SUPPLEMENTARY MATERIAL**

**Healing the Unhealable: Wharton's Jelly Stem Cell Vesicles as a Breakthrough for Feline Chronic Skin Ulcers**

**Supplementary Table S1. Individual Treatment Details and Demographics for Study Participants (n=20 cats)**

| **Cat**  **ID** | **Group** | **Signalment**  **(Age/Sex/Wt)** | **Wound**  **Etiology**  **& Location** | **Initial Area (cm²)** | **Systemic**  **Antibiotics** | **Concurrent Therapy**  **(Antipruritic/Analgesic)** | **Outcome**  **(Day 16**  **Closure)** |
| --- | --- | --- | --- | --- | --- | --- | --- |
| EV01 | EV-Treated | 7y / M / 4.5kg | Traumatic (Bite) | 5.2 | Amoxicillin- | Meloxicam 0.05 mg/kg PO q24h | 96% |
|  |  |  | Dorsal Neck |  | clavulanate |  |  |
|  |  |  |  |  | 12.5 mg/kg PO  q12h |  |  |
| EV02 | EV-Treated | 12y / F / 3.8kg | Post-surgical | 8.1 | Cefovecin | Buprenorphine 0.02 mg/kg SC q8h | 89% |
|  |  |  | Dehiscence |  | 8 mg/kg SC |  |  |
|  |  |  | Lateral Thorax |  | (Single) |  |  |
| EV03 | EV-Treated | 5y / M / 5.1kg | Pressure Sore  Ischial | 3.4 | None (No active infection) | Meloxicam 0.05 mg/kg PO q24h | 92% |
|  |  |  | Tuberosity |  |  |  |  |
| EV04 | EV-Treated | 4y / F / 3.2kg | Pruritus-related | 2.1 | Amoxicillin- | Oclacitinib 0.4 mg/kg PO q12h | 98% |
|  |  |  | Self-trauma |  | clavulanate |  |  |
|  |  |  | Pre-auricular |  | 12.5 mg/kg PO  q12h |  |  |
| EV05 | EV-Treated | 8y / M / 6.0kg | Traumatic | 6.5 | Cefovecin | Buprenorphine 0.02 mg/kg SC q8h | 91% |
|  |  |  | (Laceration) |  | 8 mg/kg SC |  |  |
|  |  |  | Forelimb |  | (Single) |  |  |
| EV06 | EV-Treated | 9y / F / 4.2kg | Traumatic (Bite) | 4.8 | Amoxicillin- | Meloxicam 0.05 mg/kg PO q24h | 94% |
|  |  |  | Flank |  | clavulanate |  |  |
|  |  |  |  |  | 12.5 mg/kg PO  q12h |  |  |
| EV07 | EV-Treated | 11y / M / 5.5kg | Pressure Sore | 2.9 | None | Meloxicam 0.05 mg/kg PO q24h | 88% |
|  |  |  | Lateral Hock |  |  |  |  |
| EV08 | EV-Treated | 3y / F / 3.5kg | Pruritus-related | 4.1 | Amoxicillin- | Cyclosporine 5 mg/kg PO q24h | 95% |
|  |  |  | Self-trauma |  | clavulanate |  |  |
|  |  |  | Cervical |  | 12.5 mg/kg PO  q12h |  |  |
| EV09 | EV-Treated | 6y / M / 7.2kg | Post-surgical | 10.4 | Cefovecin | Buprenorphine 0.02 mg/kg SC q8h | 85% |
|  |  |  | Dehiscence |  | 8 mg/kg SC |  |  |
|  |  |  | Abdominal |  | (Single) |  |  |
| EV10 | EV-Treated | 5y / F / 4.0kg | Traumatic | 3.8 | Amoxicillin- | Meloxicam 0.05 mg/kg PO q24h | 96% |
|  |  |  | (Laceration) |  | clavulanate |  |  |
|  |  |  | Tail Base |  | 12.5 mg/kg PO  q12h |  |  |
| C01 | Control | 4y / M / 5.8kg | Traumatic (Bite) | 5.5 | Amoxicillin- | Meloxicam 0.05 mg/kg PO q24h | 62% |
|  |  |  | Lumbar |  | clavulanate |  |  |
|  |  |  |  |  | 12.5 mg/kg PO  q12h |  |  |
| C02 | Control | 13y / F / 3.3kg | Post-surgical | 7.2 | Cefovecin | Buprenorphine 0.02 mg/kg SC q8h | 45% |
|  |  |  | Dehiscence |  | 8 mg/kg SC |  |  |
|  |  |  | Thorax |  | (Single) |  |  |
| C03 | Control | 8y / M / 6.1kg | Traumatic | 4.9 | Cefovecin | Buprenorphine 0.02 mg/kg SC q8h | 58% |
|  |  |  | (Laceration) |  | 8 mg/kg SC |  |  |
|  |  |  | Hindlimb |  | (Single) |  |  |
| C04 | Control | 6y / F / 4.4kg | Pruritus-related | 2.5 | Amoxicillin- | Oclacitinib 0.5 mg/kg PO q12h | 71% |
|  |  |  | Self-trauma |  | clavulanate |  |  |
|  |  |  | Face |  | 12.5 mg/kg PO  q12h |  |  |

| **Cat**  **ID** | **Group** | **Signalment**  **(Age/Sex/Wt)** | **Wound**  **Etiology**  **& Location** | **Initial Area (cm²)** | **Systemic**  **Antibiotics** | **Concurrent Therapy**  **(Antipruritic/Analgesic)** | **Outcome**  **(Day 16**  **Closure)** |
| --- | --- | --- | --- | --- | --- | --- | --- |
| C05 | Control | 10y / M / 4.9kg | Pressure Sore | 3.8 | None | Meloxicam 0.05 mg/kg PO q24h | 48% |
|  |  |  | Elbow |  |  |  |  |
| C06 | Control | 3y / F / 3.6kg | Post-surgical | 6.0 | Amoxicillin- | Meloxicam 0.05 mg/kg PO q24h | 55% |
|  |  |  | Dehiscence |  | clavulanate |  |  |
|  |  |  | Inguinal |  | 12.5 mg/kg PO  q12h |  |  |
| C07 | Control | 7y / M / 5.2kg | Traumatic (Bite) | 5.1 | Amoxicillin- | Meloxicam 0.05 mg/kg PO q24h | 65% |
|  |  |  | Shoulder |  | clavulanate |  |  |
|  |  |  |  |  | 12.5 mg/kg PO  q12h |  |  |
| C08 | Control | 9y / F / 4.1kg | Pruritus-related | 3.5 | Amoxicillin- | Cyclosporine 5 mg/kg PO q24h | 68% |
|  |  |  | Self-trauma |  | clavulanate |  |  |
|  |  |  | Neck |  | 12.5 mg/kg PO  q12h |  |  |
| C09 | Control | 5y / M / 4.7kg | Pressure Sore | 2.8 | None | Meloxicam 0.05 mg/kg PO q24h | 51% |
|  |  |  | Tarsus |  |  |  |  |
| C10 | Control | 11y / F / 3.9kg | Post-surgical | 10.0 | Cefovecin | Buprenorphine 0.02 mg/kg SC q8h | 60% |
|  |  |  | Dehiscence |  | 8 mg/kg SC |  |  |
|  |  |  | Dorsal |  | (Single) |  |  |

***Abbreviations:*** *PO: Per Os (orally); SC: Subcutaneous; q12h: Every 12 hours; q24h: Every 24 hours.*

***Note:*** *All animals received daily standard wound care consisting of cleaning with sterile saline and protective bandaging.*
